# The Impact Mechanism of Consumer’s Initial Visit to an Automobile 4S Store on Test Drive Intention: Product Aesthetics, Space Image, Service Quality, and Brand Image

**DOI:** 10.3390/bs13080673

**Published:** 2023-08-11

**Authors:** Qianling Jiang, Liyuan Deng, Chun Yang

**Affiliations:** School of Design, Jiangnan University, Wuxi 214122, China; jiangqianling@jiangnan.edu.cn (Q.J.); 8202201014@jiangnan.edu.cn (C.Y.)

**Keywords:** test drive intention, product aesthetics, space image, service quality, brand image

## Abstract

[Purpose/Significance] Under the influence of various factors such as the pandemic, oil prices, and economic conditions, the global automotive industry has entered a period of downturn. Therefore, how to activate market potential and increase consumers’ willingness to purchase cars has become an important research topic. Unlike many other products, test drives play a significant role in the car-buying process. [Method/Procedure] This study employs a questionnaire survey to explore how consumer perceptions of product aesthetics, space quality, and service quality during their initial visit to an automobile 4S store influence their test drive intention through two dimensions of brand image: symbolic and experiential. A structural equation model is used to establish a test drive intention impact model incorporating these dimensions. [Results/Conclusions] The study found that brand image, both symbolic and experiential, plays a significant mediating role in enhancing potential consumers’ test drive intentions. Space image had the most significant impact on brand image. Although product aesthetics did not directly affect brand image experientially, they remained an important factor in enhancing brand image symbolically. [Contribution/Value] The results of this study can provide insights for automotive brand managers, automobile 4S store designers, and others aiming to promote the sustainable development of automotive consumption.

## 1. Introduction

In 2019, global automotive production surpassed 1 billion [1]. However, following the outbreak of the COVID-19 pandemic, the global economy rapidly declined, resulting in a sharp drop in global automotive sales in 2020, decreasing by approximately 12.45 million compared with 2019 [2]. Even in 2022, global automotive sales remained 11% lower than pre-pandemic levels [3]. Although there has been some improvement in global automotive sales in the first quarter of 2023, the overall situation remains weak, and future sales are projected to struggle to reach previous highs [4]. This indicates that the global automotive industry entered a period of downturn after 2020 and remains entrenched in it. Even in China, the world’s largest automotive market, automotive sales face significant challenges [5]. Therefore, activating market potential and increasing consumer car-buying intentions have become critical research topics.

Automobile 4S stores, short for Automobile Sales Service Shop 4S, are automotive sales enterprises that integrate vehicle sales, spare parts, after-sales service, and customer feedback. As consumer demands evolve, consumers no longer solely focus on purchasing a single product but place greater emphasis on comprehensive and integrated service experiences. Effective, convenient, high-quality, and customer-centric services provided by 4S enterprises have become vital for increasing automotive sales and generating profits [6]. In the current period of automotive sales downturn, adjusting the operational model of automobile 4S stores to increase sales has garnered industry attention. Wu studied the sales force of 4S stores in the digital marketing department and analyzed the performance attribution of employees, providing strategies for internal quality improvement of 4S stores [7]. Huang investigated the after-sales service quality of Mercedes-Benz 4S stores and improved their brand image and customer loyalty through upgrading the existing management mode [8]. Wang et al. proposed a modified Recurrent Neural Network (RNN-2L) based on customer absence and presence behavior to help businesses identify and retain valuable but potential customers [9]. Zhou explored the intelligent service model and digital marketing system of automobile 4S stores based on big data and digital technology [10].

As automobile 4S store marketing strategies shift from simple forms to comprehensive aspects such as brand image culture and customer loyalty, the test drive experience has gradually become a new focus in automotive marketing [7]. Despite the abundance of online resources and information in the digital era, test driving remains a crucial element for consumers to gain true experience with vehicles. Through test driving, consumers can personally feel the driving performance, handling, and comfort of the vehicle, enabling them to make more accurate car purchase decisions. Therefore, test driving is an indispensable part of the car-buying decision-making process. However, few studies have focused on consumer test drivers of cars. For instance, Stryja and Satzger found that obstacles to consumers testing electric vehicles include a lack of convenience and incentives, low media exposure, limited points of contact in daily life, and age restrictions [11]. Yavorsky et al. discovered that consumer test driving plans and intentions are related to the attractiveness of the car brand, the number in 4S stores, distance, and retrieval costs [12]. Herziger and Sintov confirmed that test driving electric vehicles increases both public and private symbolic meanings, and the changes in private symbolic meanings before and after test driving affect the intention to adopt electric vehicles [13].

Automotive test driving is one of the key characteristic services offered by automobile 4S stores and a crucial prerequisite for car purchases and increasing purchase intentions. Research suggests that consumers usually visit stores and inspect products by themselves before making purchase decisions, making test driving a critical avenue for consumers to find suitable products for themselves [12]. However, there is still a gap, which may present certain obstacles for the automotive industry to emerge from its downturn, in research regarding which factors in automobile 4S stores influence and how they affect consumers’ test drive intentions. Hence, this study aims to explore how factors such as products, space, services, brand symbolic meanings, and user experiences in automobile 4S stores influence consumers’ test drive intentions in the unique environment of these stores. It also aims to investigate the underlying relationships between these factors, providing reference ideas for the improvement and optimization of automobile 4S stores to enhance test drive intentions and promote new car sales.

## 2. Research Hypotheses and Model Development

### 2.1. Brand Image and Test Drive Intention

Brand image refers to the sum of impressions consumers acquire from various sources and the views formed about a brand through brand associations [14]. Brand image is the core driver of brand assets, and the level of brand assets determines user brand loyalty [15,16], thereby directly influencing consumer behavioral intentions [17]. Brand image plays a crucial role in differentiating companies and serves as a powerful marketing tool [18]. Automobile 4S stores have significant advantages in enhancing the automotive brand and image [19], and studies have already confirmed that different automotive brand images can lead to varying degrees of increase in consumers’ test drive plans [12]. Therefore, when 4S automobile stores possess a positive brand image, consumers are more inclined to explore and engage with their automotive products, leading to the formation of test drive intentions.

#### 2.1.1. Symbolic Dimension

Symbolic meaning is a powerful driver of brand image and consumer choices [20,21]. Consumers often associate brand symbolism with their personal identities to enhance their self-esteem and social status [22], leading some consumers to prefer brands with symbolic meanings. Automobiles, as social products, already possess symbolic attributes [23]. For instance, the brand image of electric vehicles embodies symbolism related to environmental sustainability, innovative technology, and social welfare [24], reflecting three symbolic self-identities of users: environmentalists, innovators, and socially responsible citizens [25]. This enhances users’ identification and interest in test-driving electric vehicles. Long et al. also confirmed in their study that Tesla’s brand image conveys symbolic meanings associated with high technology, social status, and eco-friendly contributions, influencing consumers’ preferences and intentions for car selection and purchase [26]. Therefore, when the brand’s symbolic meaning of an automobile 4S store satisfies users’ desire for spiritual consumption, their test drive intention is likely to increase. This leads to the formation of Hypothesis 1 (H1):

**H1:**  *The symbolic dimension of brand image positively influences consumers’ test drive intentions.*

#### 2.1.2. Experiential Dimension

Brands themselves serve as a rich source of sensory, emotional, and cognitive associations, which lead to memorable and meaningful brand experiences [27]. Therefore, experiences are crucial in the process of shaping a brand. Zarantonello et al. argue that providing engaging experiences to consumers is a superior approach compared with conventional sales methods [28], and positive experiences ultimately lead to consumer brand love [29]. In the context of the experience economy, Sheng confirmed that brand experiences have the most significant impact on user behavioral intentions in the automotive industry [30]. Consequently, the quality of experiences greatly influences the interaction between users and brand enterprises. In the context of automobile 4S stores, this interaction primarily manifests through users’ participation in test-driving vehicles. Hence, the second Hypothesis (H2) of this study is:

**H2:**  *The experiential dimension of brand image positively influences consumers’ test drive intentions.*

### 2.2. Product Aesthetics

Product aesthetics typically refers to the “philosophy of beauty” associated with a product object [31], which is expressed through color, material, shape, style, and proportion [32]. As a strategic tool, product aesthetics can reinforce brand image and symbolic meaning, thereby influencing consumer beliefs. Brunner et al. confirmed in their research that under consistent design, product aesthetics positively influence the brand’s symbolic meaning and consumer brand evaluations [33]. Consistent with previous research, this study posits that in automobile 4S stores, the presence of high-quality aesthetic design in both the store and internal automotive products can enhance the brand’s symbolic meaning.

Product aesthetics represent an objective component [34], but during the interaction with users, they stimulate the senses, elicit varying degrees of behavioral, sensory, and reflective responses to the product, and trigger various subjective psychological processes [35], leading to different experiential perceptions [36]. Cunha confirmed in their study that the centrality of visual product aesthetics has a positive impact on brand experience [37]. Petruzzellis and Winer also noted that perceived aesthetics positively moderate brand experiences [38]. Therefore, this study proposes that during their visit to 4S automobile stores, users’ experiences are influenced by the unique product aesthetic features of the vehicles, further affecting their decision to explore and experience the car, such as through a test drive. These discussions lead to Hypothesis 3 (H3) and Hypothesis 4 (H4):

**H3:**  *Product aesthetics positively influence the experiential dimension of brand image.*

**H4:**  *Product aesthetics positively influence the symbolic dimension of brand image.*

### 2.3. Space Image

Automobile 4S store display spaces have always been a crucial component of automotive marketing [39]. Architectural research indicates that buildings can convey implicit symbolic meanings through the shaping of spatial exteriors and interior decorations using associations and metaphors [40]. Automobile 4S stores, as a form of architecture, often employ unique features of their automotive brand in interior space design to subliminally construct brand image and convey symbolic meanings. For instance, a study on Ferrari dealerships revealed that all furnishings inside the store were shaped like racing cars or racetrack curves, emphasizing the flow of curves. Through various images and decorations in the space, both the speed and fashion symbolism of the Ferrari brand were showcased [41]. Therefore, this study posits that the spatial image of an automobile 4S store can shape a stronger brand image’s symbolic meaning.

Furthermore, excellent spatial design and layout can influence consumers’ shopping experiences [42]. A well-designed automobile 4S store space can evoke positive emotions and create an ideal experience for users [43,44]. Yüksel et al. argued that the spatial environment significantly impacts users’ emotions, attitudes, and behaviors [45], with the atmosphere and imagery within the space designed to evoke emotional responses and influence the post-consumption experience [46]. Jun et al. confirmed in their research that creating differentiated spatial features through localization can lead to a powerful spatial experience [47]. By extension, if an automobile 4S store possesses a unique spatial image, consumers can have a more favorable brand experience within it. Based on the above discussions, Hypothesis 5 (H5) and Hypothesis 6 (H6) can be formulated:

**H5:**  *The space image of an automobile 4S store positively influences the experiential dimension of brand image.*

**H6:**  *The space image of an automobile 4S store positively influences the symbolic dimension of brand image.*

### 2.4. Service Quality

Service quality is often regarded as a crucial aspect of a company’s competitiveness, reflecting the performance of the entire service system [48]. Building brand image also involves the participation of service elements, hence the close relationship between service quality and brand image in automobile 4S stores. Nam et al. pointed out that consistency among various service elements can enhance symbolic meaning within the service industry [49]. Jiménez Barreto et al. argued that through good service quality, consumers can individually obtain the symbolic value of a brand [50]. Therefore, the overall service quality of an automobile 4S store can be improved, which can shape its brand’s symbolic meaning.

Experience is a unique part generated during the service process [51]. Service quality is often considered a precursor to customer satisfaction and behavioral intentions, which in turn directly influence the user experience [52,53]. Lee et al. confirmed in their research that improving service quality enhances users’ experiential outcomes [54]. Prentice et al. also emphasized that service quality contributes to brand experience, and in a multi-touchpoint brand experience, high-quality service during each encounter enhances customers’ experience with the brand [55]. Therefore, in an automobile 4S store, service quality directly impacts the brand’s experiential outcomes. Hence, Hypothesis 7 (H7) and Hypothesis 8 (H8) can be proposed:

**H7:**  *The service quality of an automobile 4S store triggers a more positive experiential dimension of brand image.*

**H8:**  *The service quality of an automobile 4S store triggers a more positive symbolic dimension of brand image.*

**Figure 1:**
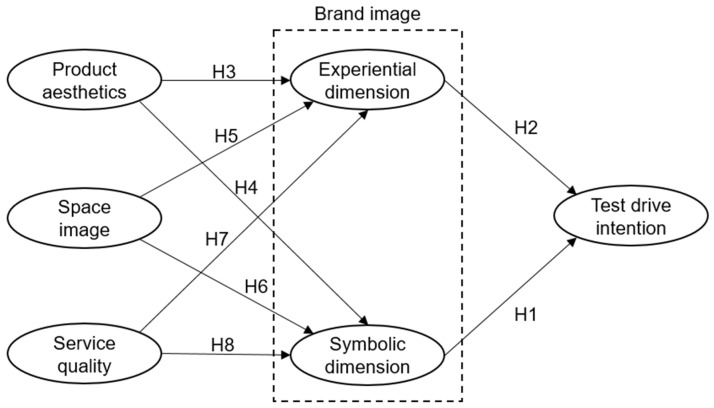
Hypothetical model.

Based on the 8 hypotheses proposed above, the theoretical model framework can be illustrated as shown in Figure 1.

## 3. Research Design and Methods

### 3.1. Questionnaire Design

To ensure the reliability of the study, the scales used in this research are derived from validated and established scales found in relevant literature. These scales were adjusted based on the specific characteristics of the research topic to create the final questionnaire. The questionnaire consists of two parts: the first part collects basic information from the respondents, while the second part measures the perceptions and behaviors of potential consumers during their first visit to a 4S car dealership, as shown in Table 1. Likert’s seven-point scales are used for the latent variables, where “1” represents “strongly disagree”, “2” represents “disagree”, “3” represents “somewhat disagree”, “4” represents “neutral”, “5” represents “somewhat agree”, “6” represents “agree”, and “7” represents “strongly agree.” Respondents are instructed to select the response that best reflects their actual experiences.

### 3.2. Data Collection

The survey for this study was conducted from July 2022 to July 2023 in Wuxi, China. The research targeted traditional household automobile brands, including four higher-end brands: BMW, Audi, Cadillac, and Lexus, as well as three more general brands: Toyota, Buick, and Volkswagen. Potential consumers who visited one of the 4S stores of these automobile brands for the first time were invited to participate in the survey. We specifically asked these potential consumers if they were familiar with the brand and if they had obtained any detailed information about the brand, including product images, from the internet. Only potential consumers who answered that they were not familiar with the brand and had not obtained any information about it from the internet were included in the survey. This also included potential consumers who had heard about these brands but did not have complete knowledge about them. All participants were asked to sign an informed consent form, and after completing the questionnaire, they were compensated accordingly for their participation.

A total of 354 questionnaires were collected, and after removing invalid questionnaires (e.g., those with inconsistent answers to trap questions or with missing responses to some items), the final valid sample size was 308. The sample size exceeded 10 times the number of items used in the analysis (18), meeting the requirements for conducting Structural Equation Modeling (SEM). Table 2 presents the demographic information of the respondents.

## 4. Data Analysis

### 4.1. Reliability Test

This study conducted a reliability analysis of each latent variable using SPSS software. The Cronbach’s alpha coefficients for each latent variable were as follows: Product aesthetics (0.818), Space image (0.761), Service quality (0.756), Symbolic (0.752), Experiential (0.831), and Test drive intention (0.750). The results indicated that the data had excellent internal consistency, and no items should be removed as doing so would result in lower reliability for the latent variables. The overall data reliability was deemed to be very good, which is considered suitable for the subsequent analysis.

### 4.2. Exploratory Factor Analysis

Exploratory factor analysis was employed in this study to examine the unidimensionality of each latent variable. Principal component analysis was conducted to extract new factors with eigenvalues greater than 1 for each latent variable. Previous research suggests that the conditions for conducting exploratory factor analysis are met when the KMO values for each latent variable are above 0.50 and the significance of the Bartlett’s sphericity test is below 0.05 [64]. The data in this study met these conditions, as shown in Table 3. All items belonging to each latent variable were included in the factor extraction process, and only one factor with an eigenvalue greater than 1 was extracted for each latent variable [65], indicating good unidimensionality.

### 4.3. Confirmatory Factor Analysis

Confirmatory factor analysis (CFA) was conducted in this study to examine the convergent and discriminant validity, as shown in Table 4. All latent variables exhibited factor loading coefficients greater than 0.5 [66], indicating strong relationships with their respective measurement items. Additionally, the average variance extracted (AVE) for each variable exceeded 0.36 [56], suggesting good convergent validity. The square root of the AVE values (diagonal elements) in Table 5 was found to be larger than the correlations between variables, indicating satisfactory discriminant validity of the measurement scale used in this study. Thus, further analysis can be conducted with confidence.

### 4.4. Model Evaluation

**Figure 2:**
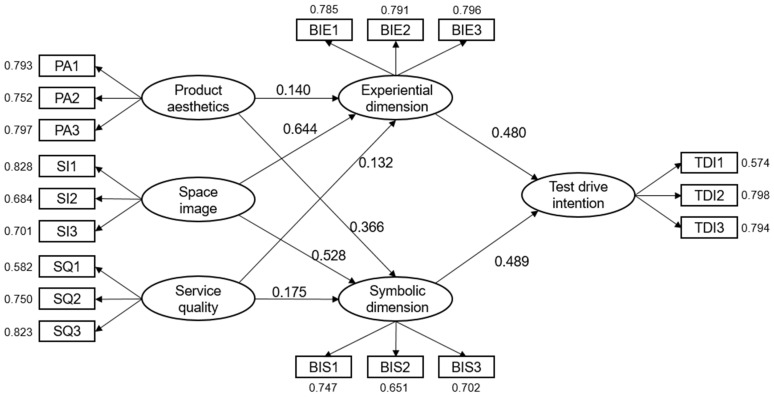
The influence between variables in the structural model.

This study conducted a path analysis of each latent variable using Amos 24 statistical software to examine their effects. The model fit indices were as follows: *χ*^2^*/df* (2.37), RMSEA (0.067), GFI (0.906), NFI (0.903), TLI (0.927), CFI (0.941), and SRMR (0.046), all falling within the ideal range. These results indicate that the model established in this study exhibits a good fit to the data. The results of the path analysis are presented in Table 6 and Figure 2. Among the eight hypotheses proposed in this study, seven are supported, while H3 is not supported, indicating that the impact of Product aesthetics on Experiential is not significant.

### 4.5. Multi-Group Comparison Analysis

Based on the different brands of 4S stores visited by the participants, this study classified the data into two groups: the regular brand group and the high-end brand group. Using Amos 24 statistical software, this study conducted a multi-group comparison of the paths in the model, and the results are shown in Table 7. The results indicate that there were no significant differences in any of the paths (z-score < 1.96). Therefore, the model proposed in this study is applicable to brands with different positionings.

## 5. Discussion

The results indicate that both the symbolic dimension of brand image and the experiential dimension of brand image have a significant impact on test drive intention (H1 and H2 are supported). Although their influence on test drive intention is similar, the symbolic dimension of brand image has a greater impact (0.489 > 0.480). The reason for this difference might be that the symbolic dimension of brand image and the experiential dimension of brand image provide consumers with different feelings and meanings. The brand image of a 4S dealership is directly linked to the symbolism of the cars it sells, while the experiences felt by consumers during the test drive may involve various aspects and may not directly reflect on the car’s features, resulting in a relatively weaker influence on test drive intention. On the other hand, the symbolic dimension of brand image not only satisfies consumers’ deeper spiritual needs but also conveys higher-level values such as social and cultural values, providing both self-identification and social recognition, status, and prestige [67]. In the context of luxury products, consumers often prefer symbolic consumption over the functionality of the product [68]. Cars have now replaced jewelry and watches as one of the key symbols of class and luxury, reflecting social status and wealth [69]. Many luxury car brands focus on building symbolic status, leading to substantial market share [70]. However, this does not mean that the brand experience is not essential. On the contrary, brand experience is a critical prerequisite for test drive intentions. Only by retaining customers through excellent brand services can opportunities be created to guide them through the test-driving experience. Additionally, some studies indicate that inherent gender differences can surpass evaluations based on external utilitarian values. For example, compared with men, women may prefer smaller, rounder, more fuel-efficient, or child-friendly cars [70,71,72].

Secondly, product aesthetics only have an impact on the symbolic dimension of brand image (H4 is supported), while their influence on the experiential dimension is not significant (not supporting H3). Product aesthetics encompass both visual aesthetics and non-visual aesthetics [34,73,74]. The perception process for non-visual aesthetic stimuli takes longer to form, while visual aesthetics have a more immediate visual appeal [75,76]. By quickly perceiving visual aesthetics, different brand images can be distinguished [77], and these distinctions between brands and the comparison of these distinctions to others contribute to the shaping, acquisition, and communication of different symbolic meanings [78,79]. In automotive 4S dealerships, consumers naturally receive and form perceptions of the symbolic dimension of brand image through visual perception of the visual product aesthetics of cars, such as their design, size, color, logo, front/side style, etc. [80]. The finding that product aesthetics do not have a significant impact on experiential aspects has also been mentioned in previous research, such as Sauer et al.’s study [73]. Due to the nature of automobiles as “black-box” products, it is challenging to visually convey the functional features related to experiential needs. As a result, consumers often tend to choose brands with visually appealing designs [6], showing a preference for brand symbolism. On the other hand, the non-visual aspects of product aesthetics, such as performance and material texture, influence the experiential aspects of brand image, but this influence requires users to interact with the automobile product to a certain extent to be formed and perceived [81]. However, such interactions are not always guaranteed to occur, resulting in the impact of product aesthetics on the experiential aspects of brand image being difficult to ascertain clearly.

Space image has a significant impact on both the symbolic and experiential dimensions of brand image (H5 and H6 are supported). Specifically, space image has a greater influence on the symbolic dimension of brand image (0.644 > 0.528), and this difference may be attributed to the characteristics of the spatial environment. As discussed earlier, the formation of the experiential dimension of brand image requires more sensory contact and interaction [82,83], while the spatial environment can be seen as a non-verbal form of communication [84,85]. It assigns meaning through “object language” and creates specific environmental cues for implicit interaction with users [86]. The space within automotive 4S dealerships provides numerous environmental cues that can evoke rich interactions and experiences [87]. Moreover, space image represents the consistent and unified expression of brand image in three-dimensional space, with the aim of generating positive brand experiences and cultivating expected brand attitudes [88]. Effective spatial feature design can leave a profound impression of the brand experience in consumers’ minds. Automobile 4S stores serve not only as spaces for purchasing vehicles but also as multifunctional exhibition spaces and public gathering areas for automobile displays, leisure activities, and automotive cultural promotion. Shaping a favorable spatial image allows users to experience more distinctive and immediate, as well as subsequent, brand experiences within the store.

Service quality also has a significant impact on the symbolic and experiential dimensions of brand image (H7 and H8 are supported). Studies have indicated the consistency between service quality and self-expression [89], as the characteristics and personality of services can influence their provision, types, and interactive effects with customers [90], thus distinguishing them from similar service providers [91]. The unique services provided by automotive 4S stores not only allow users to experience a professional and considerate experience but also convey and shape the brand’s values and symbolic meaning during the service process.

Compared with service quality and product aesthetics, space image has a greater impact on both symbolic and experiential dimensions of brand image, indicating that enhancing space image is crucial for improving brand image and subsequently influencing test drive intention. At a macro level, the space image serves as a fundamental and preliminary condition. Relevant literature has shown that the design of spatial environments can influence a person’s physiological and psychological responses [92], which are the prerequisites for perceiving brand symbolism and experiences. Various factors in the spatial environment, such as cleanliness, noise, temperature, air quality, seating comfort, and color combinations, can influence people’s decisions to enter the space and the amount of time they spend there [93,94]. Additionally, the impact of product aesthetics and service quality is actually encompassed within the broader spatial environment; physical surroundings significantly affect negativity [95]. Viewing retail products in pleasant environments leads to more positive evaluations compared with viewing the same products in unpleasant environments [96], and the same applies to the impact of service quality. At the microlevel, automobile products, due to their relatively fixed product structure, have greater constraints on the conveyance of aesthetics. Conversely, the flexibility of the spatial environment allows for more freedom [97,98], enabling significant adjustments and designs through spatial layout, configuration, and decoration, thereby creating diverse environmental cues and atmospheric spaces that better align with conveying the symbolic dimension of brand image [99]. The influence of service quality on the symbolic dimension of brand image necessitates interaction between staff and users. As evident from the aforementioned discussion, the formation and communication of symbolic brand images rely more on the uniqueness of visual aesthetics. Space image, compared with service quality, possesses a stronger visual communication effect, thus exerting a greater impact on the symbolic dimension of brand image. Regarding the impact on the experiential dimension of brand image, the environmental cues encompassed in space image directly influence users. On the other hand, the influence of product aesthetics on the experiential dimension of brand image requires events involving non-visual perception and interaction with users, which may not always occur. Therefore, space image has a greater impact on the experiential dimension of brand image compared with product aesthetics. Due to service quality’s intangible nature [100] and the fact that many services require high levels of expertise and reputation [101], they usually provide limited internal cues to form beliefs, especially during initial purchases. In such cases, consumers tend to rely on external cues, such as the physical environment, as substitute indicators for perception and inference [102]. Consequently, for consumers of automobile 4S stores, especially first-time buyers, the impact of space image on brand image experiential aspects is greater compared with service quality. The space image provides a more authentic experiential feeling, making it more influential in shaping the brand image experience.

## 6. Conclusions and Future Research

The results of this study demonstrate that the symbolic and experiential dimensions of brand image play a significant mediating role in enhancing the test drive intention of potential consumers. Both aspects have a similar and crucial influence on test drive intentions. Furthermore, space image has the most substantial impact on both the symbolic and experiential dimensions of brand image. Additionally, although product aesthetics do not affect the experiential dimension of brand image, they are still an important factor in enhancing the symbolic dimension of brand image. Lastly, although service quality has a relatively minor impact on both the experiential and symbolic dimensions of brand image, it remains significant. These findings highlight the importance of space image, service quality, product aesthetics, and brand image in enhancing the test drive intention of potential consumers.

This study provides extensive and in-depth research on the relationships among space image, service quality, product aesthetics, the symbolic dimension of brand image, the experiential dimension of brand image, and test drive intention. It offers important insights for automobile brand managers and 4S store designers. Firstly, despite the growing emphasis on service in various industries, enhancing space image remains key to improving the symbolic and experiential dimensions of brand image, even when other pre-existing factors are similar or comparable. Therefore, automobile brand managers should allocate more resources and efforts to enhancing the space image. Secondly, although product aesthetics do not directly affect the experiential dimension of brand image, their impact on the symbolic dimension is close to that of space image and significantly higher than that of service quality. Thus, product aesthetics remain a crucial indicator that cannot be ignored for enhancing the symbolic dimension of brand image. Thirdly, although service quality has the smallest impact on both the experiential and symbolic dimensions of brand image, its significance should not be overlooked. Continuing to provide high-quality services remains important.

### 6.1. Research Limitations

This study has several limitations, mainly in the following aspects: (a) Limited sample size: Due to the constraint of sample size, the results of this study may not fully represent the views and behaviors of the general consumer population. (b) Geographic limitation: The survey was conducted only in the Wuxi city of China, and as a result, the findings may be limited by the cultural, economic, and social background of this region. (c) Survey tool: This study used a questionnaire survey as the data collection method. Although questionnaires are a common research method, the results may be influenced by respondents’ subjective opinions and response biases. (d) External factors: In the real world, consumers’ test drive intentions may be influenced by various external factors, such as economic conditions, competitive brand activities, and advertising campaigns. (e) Lack of time consideration: This study only focused on the initial visit of consumers to the car 4S store and did not track changes in consumers’ test drive intentions over time.

### 6.2. Future Research

Future research can further expand the depth of research in this field in the following aspects: (a) Cross-regional studies: In order to obtain more comprehensive conclusions, future research can expand the scope of investigation and conduct studies across different regions to examine the influencing mechanisms of consumers’ test-driving intentions under different cultural, economic, and social backgrounds. Comparing data from different regions can provide a more comprehensive understanding of consumers’ behaviors and opinions in different environments. (b) Longitudinal studies: In order to understand the changes and dynamics of consumers’ test drive intentions, future research can design longitudinal studies to track and investigate consumers’ test drive intentions. Long-term data collection and analysis can reveal the trends and influencing factors of test drive intentions over time, providing a basis for automobile brand managers to develop more effective marketing strategies. (c) Multi-methods research: Combining different research methods, such as questionnaire surveys, field observations, and in-depth interviews, can improve the credibility and accuracy of the research. By collecting and analyzing data from multiple angles and dimensions, the psychological and behavioral mechanisms behind consumers’ test drive intentions can be explored in depth. (d) Exploration of external factors: Future research can investigate in more detail the influence of external factors on consumers’ test drive intentions, such as economic conditions, competitive brand activities, and advertising campaigns. In-depth research on the impact of these factors on test drive intentions can help automobile brand managers formulate more targeted marketing strategies. (e) Comparison of internal and external cues: Further explore the relative impact of internal cues (such as the internal service performance of car 4S stores) and external cues (such as the physical environment) on consumers’ test drive intentions. Comparing the importance of these two types of factors can help automobile brand managers better understand consumers’ trade-offs and priority considerations in test drive decisions. (f) New marketing models: With the development of the Internet, an increasing number of automobile brands are exploring new marketing models, such as online understanding followed by offline test drives. Future research can focus on the impact of these new marketing models on consumers’ test drive intentions and explore the influence of online-offline integrated test drive experiences on consumers’ car purchase decisions.

## Figures and Tables

**Table 1:** Part two of the questionnaire.

Construct	Coding	Item	Source
Product aesthetics	PA1	This car 4S dealership offers attractive car products.	[56,57]
	PA2	The car designs at this car 4S dealership are very appealing.	
	PA3	I like the car product designs at this car 4S dealership.	
Space image	SI1	This car 4S dealership is clean and tidy.	[58,59]
	SI2	The storefront and interior decoration of this car 4S dealership are attractive.	
	SI3	This car 4S dealership provides a variety of products.	
Service quality	SQ1	This car 4S dealership offers highly competitive services.	[60]
	SQ2	The service quality of this car 4S dealership meets my expectations.	
	SQ3	This car 4S dealership excels in various aspects of service.	
Experiential	BIE1	The products of this car brand have unique features.	[61,62]
	BIE2	Purchasing products from this car brand would bring me pleasure.	
	BIE3	The products of this car brand can fulfill my desire for a joyful life.	
Symbolic	BIS1	The products of this car brand are currently popular.	[61,62]
	BIS2	The products of this car brand can reflect my personal style.	
	BIS3	The products of this car brand symbolize my social status.	
Test drive intention	TDI1	There is a high possibility that I will test drive a car from this car brand.	[63]
	TDI2	If I have the time, I would choose to test drive a car from this car brand.	
	TDI3	I intend to test drive a car from this car brand.	

**Table 2:** Demographic information of the respondents.

	Category	Count	Ratio%
Gender	Male	144	46.75
	Female	164	53.25
Age	18–25	12	3.90
	26–34	186	60.39
	35–54	102	33.12
	55–64	8	2.60
Education background	High school or technical secondary school and below	31	10.06
	Undergraduate or junior college	226	73.38
	Graduate and above	51	16.56
Occupation	Student	26	8.44
	Private-owned enterprise	60	19.48
	National-capital enterprise	69	22.40
	Foreign-capital enterprise	55	17.86
	Public service organization	34	11.04
	Public servant	64	20.78
Brand	Toyota	67	21.75
	Volkswagen	63	20.46
	Buick	62	20.13
	Audi	49	15.91
	BMW	38	12.34
	Cadillac	26	8.44
	Lexus	3	0.97

**Table 3:** The results of exploratory factor analysis.

Construct	KMO	Bartlett Sphere Test	Item	Commonality	Factor Loading	Eigenvalue	Total Variance Explained %
Product aesthetics	0.720	0.000	PA1	0.739	0.860	2.219	73.953
			PA2	0.725	0.852		
			PA3	0.754	0.868		
Space image	0.697	0.000	SI1	0.740	0.860	2.092	69.735
			SI2	0.665	0.815		
			SI3	0.688	0.829		
Service quality	0.661	0.000	SQ1	0.555	0.745	2.017	67.244
			SQ2	0.721	0.849		
			SQ3	0.741	0.861		
Experiential	0.722	0.000	BIE1	0.726	0.852	2.252	75.077
			BIE2	0.756	0.870		
			BIE3	0.770	0.878		
Symbolic	0.688	0.000	BIS1	0.680	0.825	2.008	66.934
			BIS2	0.631	0.794		
			BIS3	0.697	0.835		
Test drive intention	0.664	0.000	TDI1	0.567	0.753	2.035	67.835
			TDI2	0.751	0.867		
			TDI3	0.717	0.847		

**Table 4:** CFA results of the measurement model.

Construct	Items	Unstandardized	Standardized	S.E.	*p*-Value	AVE	CR
		Factor Loading	Factor Loading				
Product aesthetics	PA1	1	0.795	-	-	0.610	0.824
	PA2	1.215	0.751	0.090	0.000		
	PA3	1.193	0.796	0.083	0.000		
Space image	SI1	1	0.828	-	-	0.55	0.784
	SI2	1.160	0.685	0.091	0.000		
	SI3	0.798	0.703	0.060	0.000		
Service quality	SQ1	1	0.583	-	-	0.526	0.766
	SQ2	1.433	0.755	0.157	0.000		
	SQ3	1.543	0.817	0.167	0.000		
Symbolic	BIS1	1	0.752	-	-	0.506	0.754
	BIS2	0.877	0.66	0.078	0.000		
	BIS3	1.054	0.718	0.086	0.000		
Experiential	BIE1	1	0.784	-	-	0.627	0.834
	BIE2	0.885	0.794	0.062	0.000		
	BIE3	1.051	0.797	0.073	0.000		
Test drive intention	TI1	1	0.571	-	-	0.532	0.769
	TI2	2.221	0.789	0.224	0.000		
	TI3	2.422	0.804	0.242	0.000		

**Table 5:** Discriminant validity for the measurement model.

	PA	SI	SQ	BIS	BIE	TDI
Product aesthetics	**0.781**					
Space image	0.681	**0.742**				
Service quality	0.337	0.416	**0.725**			
Symbolic	0.618	0.672	0.432	**0.711**		
Experiential	0.687	0.678	0.441	0.646	**0.792**	
Test drive intention	0.634	0.683	0.485	0.708	0.652	**0.729**

Note: The items on the diagonal in bold represent the square roots of the AVE.

**Table 6:** Regression coefficients.

Relationship	UnStd.	Std.	S.E.	*p*-Value	Hypotheses	Support
PA→BIE	0.181	0.140	0.182	0.317	H3	No
PA→BIS	0.337	0.366	0.114	0.003	H4	Yes
SI→BIE	0.765	0.644	0.190	0.000	H5	Yes
SI→BIS	0.443	0.528	0.116	0.000	H6	Yes
SQ→BIE	0.212	0.132	0.106	0.045	H7	Yes
SQ→BIS	0.199	0.175	0.068	0.004	H8	Yes
BIE→TI	0.216	0.480	0.048	0.000	H2	Yes
BIS→TI	0.312	0.489	0.068	0.000	H1	Yes

**Table 7:** Multi-Group comparison results.

Relationship	Regular Brand Group		High-End Brand Group		z-Score
	Estimate	*p*-Value	Estimate	*p*-Value	
PA→BIE	0.346	0.140	−0.096	0.823	1.716
PA→BIS	0.354	0.014	0.721	0.009	−0.902
SI→BIE	0.522	0.076	1.007	0.001	0.135
SI→BIS	0.362	0.043	0.261	0.106	−0.420
SQ→BIE	0.237	0.148	0.384	0.022	0.629
SQ→BIS	0.222	0.028	0.261	0.016	0.264
BIE→TI	0.175	0.003	0.285	0.000	1.143
BIS→TI	0.395	0.000	0.211	0.028	−1.373

## Data Availability

The data presented in this study are available on request from the corresponding author.
